# Comparative genetic analysis of the 45S rDNA intergenic spacers from three *Saccharum* species

**DOI:** 10.1371/journal.pone.0183447

**Published:** 2017-08-17

**Authors:** Yongji Huang, Fan Yu, Xueting Li, Ling Luo, Jiayun Wu, Yongqing Yang, Zuhu Deng, Rukai Chen, Muqing Zhang

**Affiliations:** 1 Key Lab of Sugarcane Biology and Genetic Breeding, Ministry of Agriculture, Fujian Agriculture and Forestry University, Fuzhou, China; 2 Guangdong Key Laboratory of Sugarcane Improvement and Biorefinery, Guangzhou, China; 3 Guangdong Provincial Bioengineering Institute, Guangzhou Sugarcane Industry Research Institute, Guangzhou, China; 4 Guangxi Collaborative Innovation Center of Sugar Industries, Guangxi University, Nanning, China; National Cheng Kung University, TAIWAN

## Abstract

The 45S ribosomal DNA (rDNA) units are separated by an intergenic spacer (IGS) containing the signals for transcription and processing of rRNAs. For the first time, we sequenced and analyzed the entire IGS region from three original species within the genus *Saccharum*, including *S*. *spontaneum*, *S*. *robustum*, and *S*. *officinarum* in this study. We have compared the IGS organization within three original species of the genus *Saccharum*. The IGS of these three original species showed similar overall organizations comprised of putative functional elements needed for rRNA gene activity as well as a non-transcribed spacer (NTS), a promoter region, and an external transcribed spacer (ETS). The variability in length of the IGS sequences was assessed at the individual, intraspecies, and interspecies levels of the genus *Saccharum*, including *S*. *spontaneum*, *S*. *robustum*, and *S*. *officinarum*. The ETS had greater similarity than the NTS across species, but nevertheless exhibited variation in length. Within the IGS of the *Saccharum* species, base substitutions and copy number variation of sub-repeat were causes of the divergence in IGS sequences. We also identified a significant number of methylation sites. Furthermore, fluorescent *in situ* hybridization (FISH) co-localization of IGS and pTa71 probes was detected on all representative species of the genus *Saccharum* tested. Taken together, the results of this study provide a better insight into the structure and organization of the IGS in the genus *Saccharum*.

## Introduction

Sugarcane, belonging to the genus *Saccharum* in the family *Poaceae* and the tribe *Andropogoneae*, is mainly cultivated in tropical and subtropical regions of both hemispheres [1]. Earlier classification of species within this genus has been based on morphological, cytological, and biochemical characteristics [2]. However, the taxonomy within the genus *Saccharum* remains controversial. Over the past two decades, molecular cytogenetics and markers have contributed to inference of the phylogenetic relationship of the species within the genus *Saccharum* [3–7], and therefore the classification of the species within this genus has gradually been elucidated. The currently accepted taxonomy for the genus *Saccharum* comprises six species, including *Saccharum officinarum*, *Saccharum robustum*, *Saccharum spontaneum*, *Saccharum sinense*, *Saccharum barberi*, and *Saccharum edule* [8]. Of these, *S*. *spontaneum* and *S*. *robustum* are considered to be wild species [9]. *S*. *spontaneum* is widely recognized as the most primitive species within the genus *Saccharum*, whereas *S*. *robustum* has been postulated to be the progenitor of the high sugar content species, *S*. *officinarum* [10]. These three species represent the original species within the genus *Saccharum*. The chromosome number of *S*. *spontaneum* ranges from 2*n* = 40–128 with a basic chromosome number *x* = 8, while that of *S*. *officinarum* and *S*. *robustum* is constant at 2*n* = 80 with a basic chromosome number *x* = 10 [6]. The remaining three species are thought to have interspecific or intergeneric origins. *S*. *sinense* and *S*. *barberi* are believed to be derived from naturally interspecific hybridization between *S*. *officinarum* and *S*. *spontaneum* [7]. *S*. *edule*, which is characterized by its abortive flowers, is thought to have arisen from intergeneric crosses between *S*. *officinarum* or *S*. *robustum* and a related genus, or derived from *S*. *robustum* [9, 11–14].

**Fig 1:**
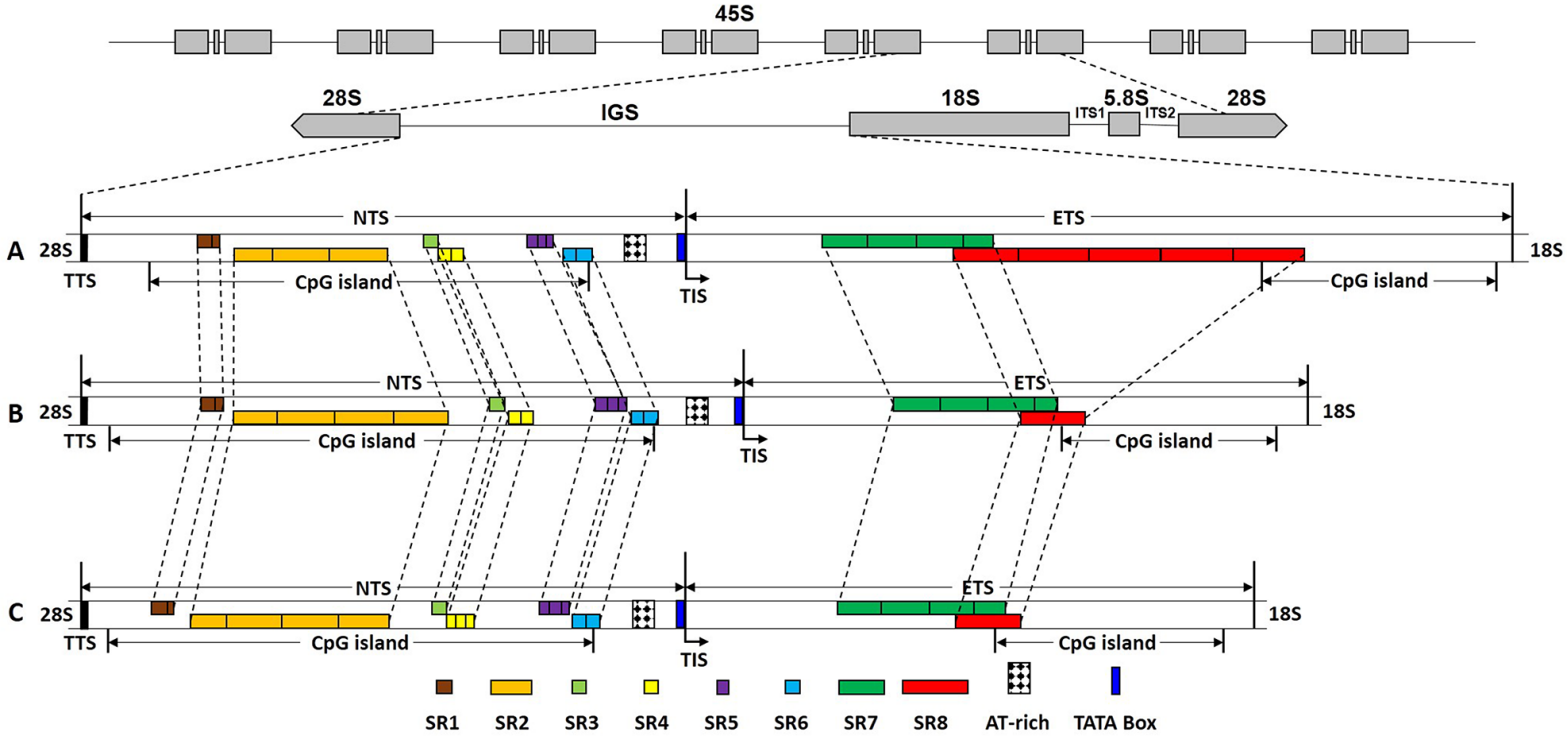
Canonical structural organization of the IGS regions in (A) Yunnan82-114 (*S*. *spontaneum*), (B) 51NG3 (*S*. *robustum*) and (C) Luohanzhe (*S*. *officinarum*). IGS: intergenic spacer; TTS: transcription termination site; NTS: non-transcribed spacer; TIS: transcription initiation site; ETS: external transcribed sequence; SR: sub-repeat. CpG island: cytosine-guanine island.

In higher plants, nuclear ribosomal DNA (rDNA) belongs to two universal multiple tandem repeats gene families, the minor 5S rDNA and major 45S rDNA [15]. The minor 5S rDNA units consist of a 5S rRNA gene region transcribed by RNA polymerase III, a conserved transcribing region of 120 bp, and a variable non-transcribed spacer (5S rDNA NTS, hereafter referring to as the non-transcribed region in the 5S rDNA unit). The 5S rDNA consists of multiple tandemly arrayed repeats at one or several chromosomal locations throughout the genome. On the other hand, the major 45S rDNA units are located at the nucleolar organizer regions (NORs) with a high copy number and are arranged in tandem at one or several sites per haploid genome. Each 45S rDNA repeated unit is composed of the coding region for the 18S, 5.8S, and 25S ribosomal RNA (rRNA) genes separated by two internal transcribed spacers (ITS1 and ITS2). Moreover, each transcribed unit is separated by a large non-transcribed intergenic spacer (IGS). The IGS can be subdivided into the non-transcribed region (NTS, hereafter referring to the non-transcribed region in 45S rDNA unit, but not the non-transcribed region in 5S rDNA unit), and the external transcribed spacer (ETS) (Fig 1) [15]. The 45S rRNA genes, which are transcribed by RNA polymerase I, are highly conserved throughout plants, animals, and fungi. However, the functional role of the IGS region is conserved and contains the RNA polymerase I promoter and other essential regulatory elements, such as sub-repeat (SR) elements, a transcription initiation site (TIS), a transcription termination site (TTS), and enhancers.

The 45S rDNA undergoes a peculiar pattern of evolution referred to as concerted evolution [16, 17]. This is an evolution process that homogenizes all of the 45S rDNA repeat units within the array and fixes the 45S rDNA repeat units during chromosomal recombination within a sexually reproducing species. Despite the presence of concerted evolution leading to the slowly evolving 45S rRNA gene, the noncoding IGS sequence changes rapidly. Variations in both length and organization of the IGS region have been commonly observed among individuals as well as intraspecies and interspecies of several species. Notably, it has been recently reported that IGS regions in *Medicago arborea* showed heterogeneity in a single 45S rDNA locus [18]. Variability in the IGS region is thought to be the result of diversity in the numbers of SRs generated by unequal crossing over [19]. Moreover, in higher eukaryotes, DNA is methylated exclusively at 5-methyl-cytosine to guanosine in the cytosine-phosphate-guanosine (CpG) dinucleotides [20]. This modification is one of the major events causing gene repression, especially when involving the areas of the GC-rich base composition and high density of CpG dinucleotides known as CpG islands. In fact, the 5S rDNA, 45S rDNA, and IGS in eukaryotes seem to be also highly methylated [21–23].

In contrast to the evolutionary conservation in 45S and 5S rRNA genes, the spacers (ITS, IGS, and 5S rDNA NTS) are highly variable both in length and sequence [24]. These spacers are valuable for phylogenetic inference at low taxonomic levels because they show high nucleotide substitution rates and low intragenomic sequence heterogeneity [25, 26]. Indeed, the spacers have been widely used for resolving phylogenetic relationships over the past three decades [27–30]. The vast majority of studies using rDNA sequences for phylogeny and taxonomy have focused on the 45S rDNA ITS or 5S rDNA NTS. Conversely, the IGS region has been poorly investigated in the taxonomy [25, 26]. Owing to the apparent lack of selective constraints, the IGS region is considered to be the most rapidly evolving spacer region within the rDNA array and displays the highest variability. Therefore, it is suitable for phylogenetic analyses of closely related species.

IGS region plays a vital role in the regulation of 45S rDNA transcription because it is a complex modular unit containing consensus molecular structures for regulatory and processing signals of 45S rRNAs [24]. In fact, characterization of the structure and organization of IGS region could provide insight into the control of gene expression of 45S rRNA genes. So far, the sequence data of IGS region have been available for several plants of different families, including *Poaceae*, *Brassicaceae*, *Cucurbitaceae*, *Solanaceae* and *Fabaceae* [25–30]. However, to our knowledge, the comparative analysis of the IGS sequence, including the structural and functional importance of its constituents, has never been attempted among three original species of the genus *Saccharum*, including *S*. *spontaneum*, *S*. *robustum*, and *S*. *officinarum*. In the present study, we characterized the complete sequence of the IGS region in the genus *Saccharum* for the first time. The objectives of the present study were as follows: (1) to understand the organization of the IGS sequence among the three original species within the genus *Saccharum*; (2) to assess the individual, intraspecies, and interspecies heterogeneity in the IGS sequence; and (3) to investigate the chromosomal location between 45S rDNA and IGS in the three original species using fluorescence in situ hybridization (FISH).

## Materials and methods

### Plant material and DNA extraction

All plant materials used in this study were grown in the nursery from Sugarcane Research Institute, Yunnan Academy of Agricultural Sciences as stated in Table 1. At present, these plant materials were conserved in the China National Nursery of Sugarcane Germplasm Resources, which was built by China's Ministry of Agriculture in Kaiyuan city, Yunan province in 1995. We selected five genotypes within each original species, including *S*. *spontaneum*, *S*. *robustum*, and *S*. *officinarum*. In total, 15 genotypes of the genus *Saccharum* were selected. Leaf tissues of the above materials were ground in liquid nitrogen and stored at -80°C. Total genomic DNA was extracted from young leaves following CTAB methodology [31].

**Table 1:** The 15 genotypes of the genus *Saccharum* used in this study.

No.	Accession	Species	Ploidy level	Chromosome number
1	Yunnan82-215	*S*. *spontaneum*	Octaploid (8*x*)	2*n* = 64
2	Yunnan83-201	*S*. *spontaneum*	Nonaploid (9*x*)	2*n* = 72
3	Yunnan82-114	*S*. *spontaneum*	Decaploid (10*x*)	2*n* = 80
4	Fujian Huian	*S*. *spontaneum*	Dodecaploid (12*x*)	2*n* = 96
5	Fujian89-1-19	*S*. *spontaneum*	Tridecaploid (13*x*)	2*n* = 104
6	51NG3	*S*. *robustum*	Octaploid (8*x*)	2*n* = 80
7	57NG208	*S*. *robustum*	Octaploid (8*x*)	2*n* = 80
8	Daye	*S*. *robustum*	Octaploid (8*x*)	2*n* = 80
9	51NG63	*S*. *robustum*	Octaploid (8*x*)	2*n* = 80
10	NG77-004	*S*. *robustum*	Octaploid (8*x*)	2*n* = 80
11	Badila	*S*. *officinarum*	Octaploid (8*x*)	2*n* = 80
12	Nanjian Guozhe	*S*. *officinarum*	Octaploid (8*x*)	2*n* = 80
13	Luohanzhe	*S*. *officinarum*	Octaploid (8*x*)	2*n* = 80
14	Crystallina	*S*. *officinarum*	Octaploid (8*x*)	2*n* = 80
15	Vietnam Niuzhe	*S*. *officinarum*	Octaploid (8*x*)	2*n* = 80

### TA cloning and DNA sequencing

Polymerase chain reaction (PCR) amplification was carried out using a pair of universal plant primers, IGS P3 (TTGCTGCCACGATCCACTGAG) and IGS P4 (CTACTGGCAGGATCAACCAGG) [32], which were designed according to the the conserved regions of 18S rDNA and 25S rDNA, on a Veriti 96-Well Thermal Cycler. A 50 μl reaction was performed by adding 50 ng of template DNA, 25 μl of 2 × GC Buffer I (5 mM Mg^2+^ plus), 8 μl of dNTP Mixture (2.5 mM), 20 μM of each primer, and 2.5 U of LA Taq polymerase (TaKaRa LA TaqTM, Takara Biotechnology Inc.). PCR amplification conditions were as follows: one cycle of denaturing at 95°C for 15 min, 35 cycles of denaturing at 95°C for 1 min, annealing at 58°C for 1 min, and extension at 72°C for 3 min, and final extension at 72°C for 10 min and storing at 4°C. The PCR products within a single band were purified using the QIAquick Gel Extraction Kit (Qiagen Inc.) and ligated to the pMD19-T-vector (Qiagen Inc.). The ligated product was transformed in *E*. *coli* DH 5*α* and plated onto the Amp/LB/X-gal/IPTG plates. The positive white colonies were further grown for plasmid isolation. Plasmid DNA was purified using a Plasmid Mini kit I (200) (OMEGA) and quantified. In each plant material of the 15 genotypes, eight positive plasmids from the single bacterial transformed colony were further analyzed by PCR using the specific primers of IGS sequence (IGS P3 and IGS P4). The PCR product showed the presence of DNA fragment of the expected size. In 15 genotypes of the genus *Saccharum*, eight positive plasmids for each genotype were sequenced using the Sanger sequencing method by Beijing Genomics Institute Co. Ltd (Shenzhen, China). DNA sequencing was performed using a primer walking strategy, whereby vector-specific (M13F and M13R) and internal primers were used to generate partial sequences (S1 Table). This was repeated until sequences were obtained that overlapped in the forward and reverse directions. The IGS sequences are deposited in the Genbank database under accession numbers KX254591-KX254605.

### Sequence analysis

The IGS sequence alignment was performed using the program ClustalW2 followed by manual adjustments [33, 34]. Sequence data were edited to remove the sequences at the 3′ end of 18S rDNA and the 5′ end of 25S rDNA. Alignment of the IGS sequences for all 15 plant materials using Geneious R7 [35]. Potential TATA box was found by the value of score computed by the TATA box weight matrix using Proscan 1.7 [36]. The multi-dot-matrix plot (MDMP) analysis was performed using the LBDot [37]. We used the Tandem Repeat Finder (TRF) program, which identified the SRs, and made manual adjustments using the BioEdit Sequence Alignment Editor [38]. Direct repeats and inverted repeats were detected and dot matrix analysis was performed using Unipro UGENE [39]. CpG islands were identified by CpGPlot [40].

The genetic distances among three different species were calculated according to Kimura 2-Parameter model using MEGA6.06 software. Phylogenetic analyses were performed by the maximum-likelihood (ML) and the Neighbor-joining (NJ) method according to the Kimura 2-Parameter model using molecular evolutionary genetics analysis program MEGA6, and all branches were evaluated with 1000 bootstrap replications [48].

### FISH

The chromosomal preparations and the FISH technique were conducted as described in D'Hont et al [6]. Root tips were obtained from Yunnan82-114 (*S*. *spontaneum*), 51NG3 (*S*. *robustum*), and Luohanzhe (*S*. *officinarum*). The IGS labeled by nick translation with biotin-dUTP (Roche, Switzerland) was simultaneously hybridized with pTa71, and a 9-kb fragment of rDNA from wheat was also labeled by nick translation with digoxigenin on metaphase chromosomes of the above three materials [41]. High stringency conditions of post-hybridization washes were carried out with 2 × SSC for 5 min at 42°C, a second wash in 50% formamide, 2 × SSC, pH 7.0, for 3 × 5 min at 42°C, followed by a rinse in 2 × SSC for 5 min at room temperature and a final wash in 0.1 × SSC for 3 × 5 min at 55°C. Low stringency conditions were performed in 1 × SSC for 30 min at 37°C. Chromosomes was counterstained with 4′, 6-diamidino-2-phenylindole (DAPI) in a Vectashield anti-fade solution (Vector Laboratories, Burlingame, CA). Measurements of the IGS and pTa71 fluorescent signal intensity were performed in at least in ten cells (from two different individuals) using the AxioVision measurement module of the AxioScope A1 Imager fluorescent microscope (Zeiss, Germany).

## Results

### IGS variants within individual, intraspecies, and interspecies heterogeneity

To characterize IGS sequences, IGSs from the three original species, including *S*. *spontaneum*, *S*. *robustum*, and *S*. *officinarum*, were cloned and sequenced (Table 1). The sequencing results of the entire IGS sequence revealed that variable sizes of the IGS were present in each genotype (S2 Table), which represented individual, intraspecific, and interspecific polymorphisms in the length of the IGS sequence. In eight sequences per genotype, the longest sequence had the conserved sequence to the other seven sequences, so the longest sequences per genotype was just deposited into the GenBank database. Comparisons of the IGS sequences from each level demonstrated that the individual level had the highest similarity and the interspecies level had the lowest similarity. At the intraspecies level of the genus *Saccharum*, comparisons of the IGS sequence of *S*. *robustum* and *S*. *officinarum* revealed a mean value of 87.86% of similarity and a mean GC content of 65.8%. The sizes of the IGS sequences ranged from 2592–2862 bp and 2590–2737 bp in *S*. *robustum* and *S*. *officinarum*, respectively. This result suggested that the IGS sequences of *S*. *robustum* and *S*. *officinarum* had few differences in length and a relatively low level of sequence heterogeneity (S2 Table). On the other hand, five *S*. *spontaneum* IGS clones showed some heterogeneity in length (from 2783 to 3340 bp), a mean value of 78.70% in similarity, and a mean GC content of 64.4%. This result demonstrated the high level of sequence divergence in the five *S*. *spontaneum*.

Due to heterogeneity in the length of IGSs in the three original species at the interspecies level of the genus *Saccharum*, the IGS sequences from Yunnan82-114 (*S*. *spontaneum*), 51NG3 (*S*. *robustum*), and Luohanzhe (*S*. *officinarum*) were the longest sequences in each species studied. Therefore, we chose the longest IGS sequence as a representative to outline the canonical structural organization of the IGSs from these three species of the genus *Saccharum* (S2 Table). The detailed analysis of the molecular structure of IGS sequences of these three species contains six distinct regions: NTS, ETS, SR, TTS, TIS, and a CpG island (Fig 1).

We found that all the different IGS variants from each genotype shared similar structural, regulatory and functional motifs, indicating all the different IGS variants had the necessary key elements to be functional. In addition, the differences between the different IGS variants were due to the presence or absence of sub-repeat motifs. Although it did not contradict the fact that a higher IGS diversity was present in the three species, eight sequenced clones within each genotype were enough to explore the sequence heterogeneity as well as the structure and organization of IGS region based on the two above mentioned facts.

### Functional elements and domains

#### Beginnings and ends of transcripts

**Fig 2:**
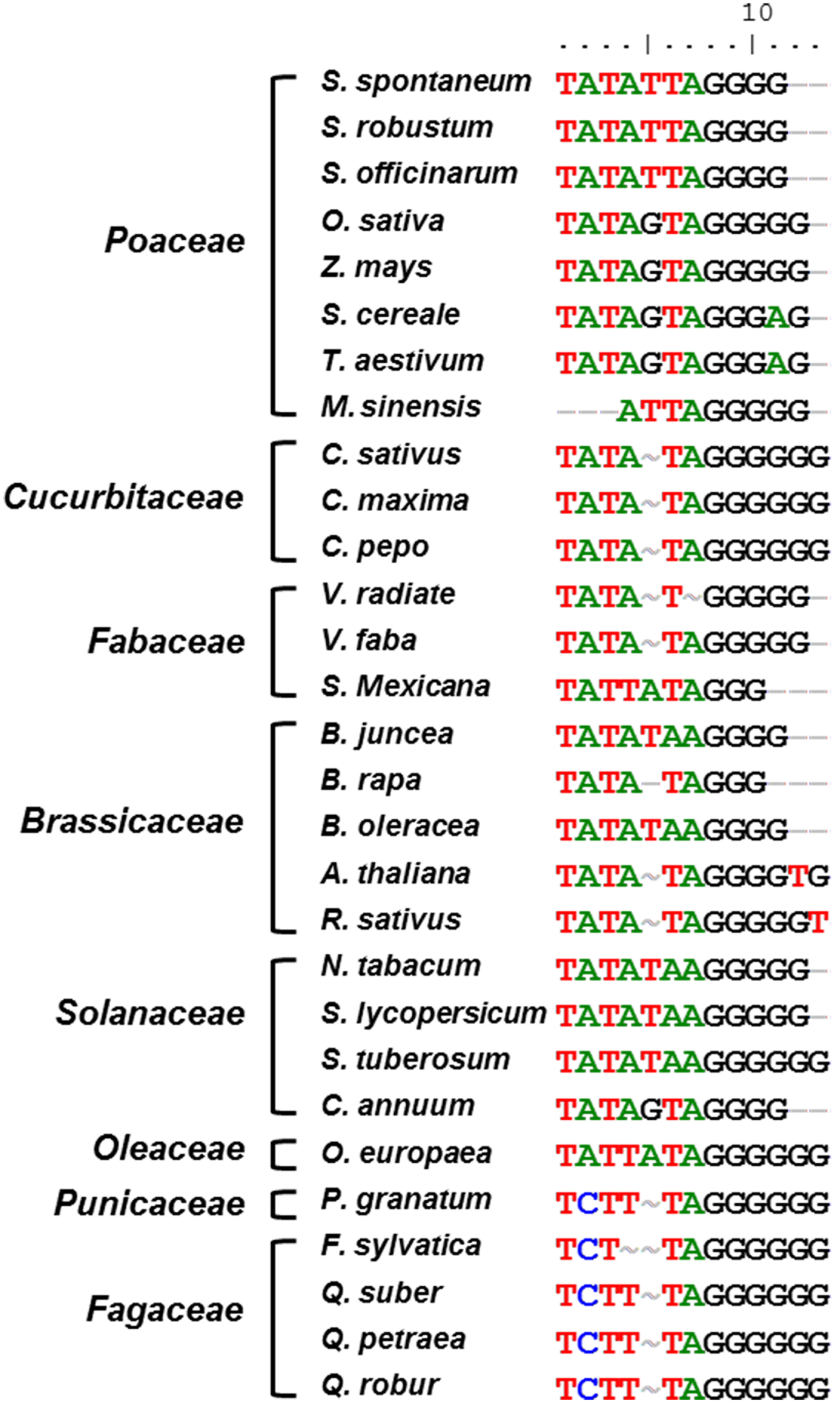
Comparison of putative TIS of different plants. The respective accession numbers: Yunnan82-114 (*S*. *spontaneum*) KX254601; 51NG3 (*S*. *robustum*) KX254591; Luohanzhe (*S*. *officinarum*) KX254598; *O*. *sativa* X54194; *Z*. *mays* X03990; *S*. *cereale* M37231; *T*. *aestivum* AJ315040; *M*. *sinensis* AJ238126; *C*. *sativus* X51542; *C*. *maxima* M28700; *C*. *pepo* X55960; *V*. *radiata* X17211; *V*. *faba* X16615; *S*. *mexicana* AJ489509; *B*. *juncea* X73032; *B*. *rapa* S78172; *B*. *oleracea* X60324; *A*. *thaliana* X52631; *R*. *sativus* Z11677; *N*. *tabacum* Y08422; *S*. *lycopersicum* AY366528; *S*. *tuberosum* AF464863; *C*. *annuum* HM352915; *O*. *europaea* AJ865373; *P*. *granatum* JX121275; *F*. *sylvatica* KC700362; *Q*. *suber* AY428812; *Q*. *petraea* EU555524; *Q*. *robur* EF208969.

Unlike the ITS region, the IGS is a non-transcribed region of rDNA. Nevertheless, it contains several highly conserved *cis*-acting functional elements [42]. To investigate the IGS sequences for functional elements and domains, we aligned the IGSs from these three species with the transcription intiation regions from of other species, including *Poaceae*, *Cucurbitaceae*, *Fabaceae*, *Brassicaceae*, *Solanaceae*, *Oleaceae*, *Fagaceae*, and *Punicaceae* [32, 43]. We found a putative TIS (TATATT**A**GGGG) for RNA polymerase I, which was in agreement with reports of TIS in other plants. The initiation site of RNA transcript at +1 position is the A residue at the middle position of the IGS region. Interestingly, adenine was present at the TIS in a majority of the higher plants, with the exception of *V*. *radiata* in the family *Fabaceae*. A putative TATA box and polyguanine sequence were generally present next to the TIS in most plants mentioned above (Fig 1). Similar to the families of *Poaceae*, *Cucurbitaceae*, *Fabaceae*, *Brassicaceae*, and *Solanaceae*, all of them shared a canonical TATA box (Fig 2). This is in agreement with most previous reports for other IGS sequences. However, *Miscanthus sinensis* (*M*. *sinensis*) in the family *Poaceae* lacked a typical TATA box [44]. In contrast to the presence of a TATA-containing sequence in the above plants, sequences lacking TATA adjacent to the TIS were flanked by pyrimidine bases in the families *Fagaceae* and *Punicaceae* [23, 43]. In addition, a unique AT-rich region that may function as an enhancer of transcription was present upstream of the TIS (Fig 1). Adjacent to the 5' end of the IGS, a typical pyrimidine rich motif of 14 bp (CCCTCCCCCCCTCC) was identified for RNA polymerase I as a putative TTS. This motif has been suggested to play a role in the finalization of rRNA transcription as a TTS.

#### NTS and ETS

The region between the 3' end of the 25S rRNA gene and the putative TIS represents the NTS, whereas the ETS was found to be located between the putative TIS and the beginning of the 18S rRNA gene (Fig 1). Due to lower functional constraints, the sequences of the NTS showed high divergence. In contrast, the ETS region has better conserved nucleotide sequences than the NTS despite having different lengths among the three species. The length differences of NTS and ETS regions among the three species were mainly due to insertions or deletions.

### CpG islands and methylation sites in the IGS sequence

Among these three species, two CpG islands were detected in NTS and ETS, respectively. However, the length of the first CpG island was longer than that of the second one. For example, the first CpG island was 667–1112 bp in length downstream from the TTS, while the second one was 534–735 bp in length downstream from the TIS, which was located just after the putative TIS (Fig 1 and S3 Table). Most of the CpG islands lie further away from the TIS [45]. It is worth noting that the IGS sequence includes a large number of GCGC and CCGG motifs that were irregularly distributed along the entire sequence (Table 2). The presence of a large number of the methylatable motifs has also been reported in other studies [46–48].

**Table 2:** Methylation sites in the IGS sequence.

No.	Genotype	Species	Ploidy level	Chromosome number	No. of methylation sites	
					GCGC	CCGG
1	Yunnan82-215	*S*. *spontaneum*	Octaploid (8*x*)	2*n* = 64	21	26
2	Yunnan83-201	*S*. *spontaneum*	Nonaploid (9*x*)	2*n* = 72	19	26
3	Yunnan82-114	*S*. *spontaneum*	Decaploid (10*x*)	2*n* = 80	16	24
4	Fujian Huian	*S*. *spontaneum*	Dodecaploid (12*x*)	2*n* = 96	17	26
5	Fujian89-1-19	*S*. *spontaneum*	Tridecaploid (13*x*)	2*n* = 104	22	29
6	51NG3	*S*. *robustum*	Octaploid (8*x*)	2*n* = 80	25	33
7	57NG208	*S*. *robustum*	Octaploid (8*x*)	2*n* = 80	26	30
8	Daye	*S*. *robustum*	Octaploid (8*x*)	2*n* = 80	23	25
9	51NG63	*S*. *robustum*	Octaploid (8*x*)	2*n* = 80	23	26
10	NG77-004	*S*. *robustum*	Octaploid (8*x*)	2*n* = 80	26	30
11	Badila	*S*. *officinarum*	Octaploid (8*x*)	2*n* = 80	23	31
12	Nanjian Guozhe	*S*. *officinarum*	Octaploid (8*x*)	2*n* = 80	22	26
13	Luohanzhe	*S*. *officinarum*	Octaploid (8*x*)	2*n* = 80	22	27
14	Crystallina	*S*. *officinarum*	Octaploid (8*x*)	2*n* = 80	23	32
15	Vietnam Niuzhe	*S*. *officinarum*	Octaploid (8*x*)	2*n* = 80	23	31

### Sub-repeat (SR) regions

**Fig 3:**
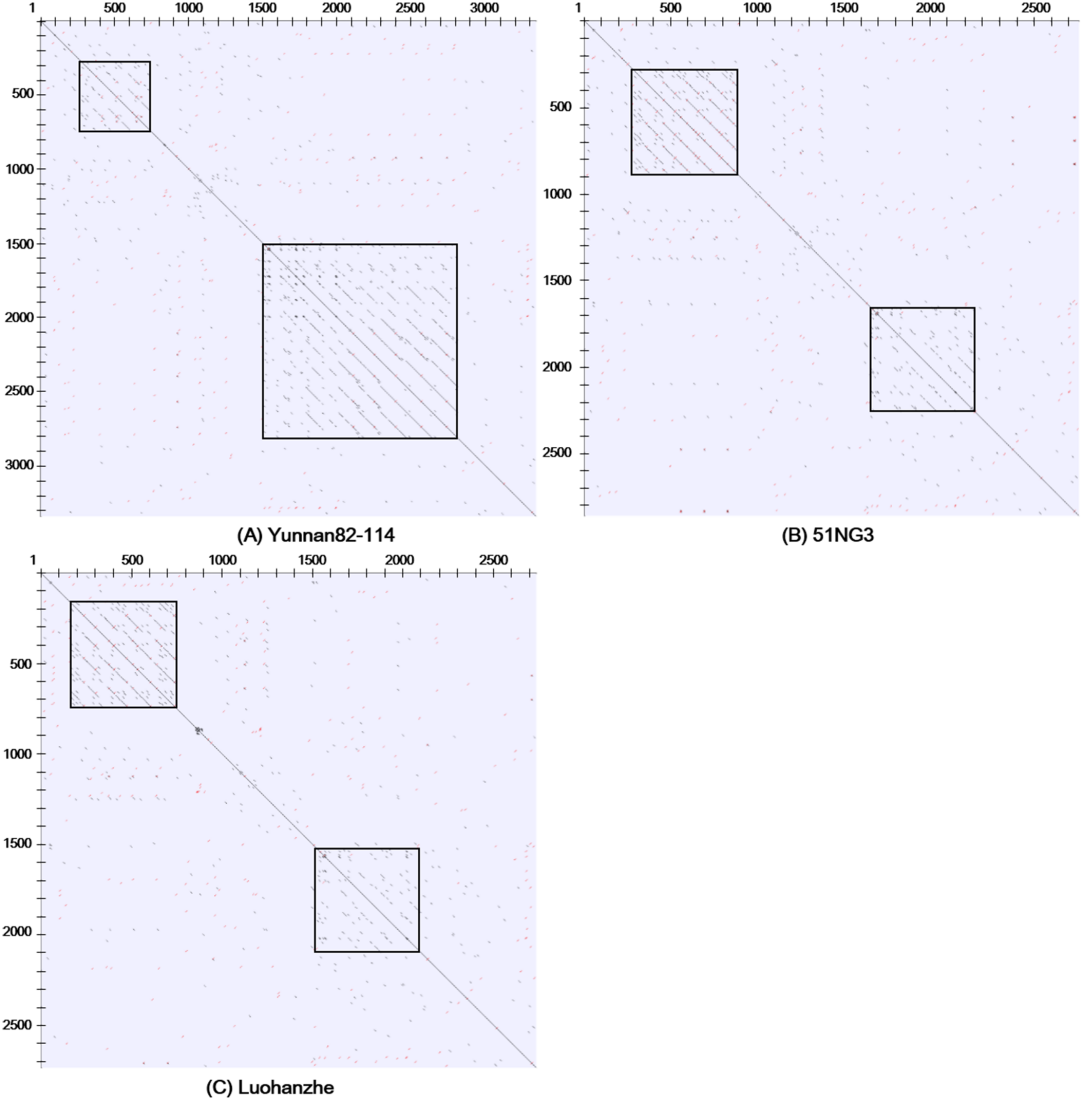
Dot matrix plot of IGS spacers in (A) Yunnan82-114, (B) 51NG3 and (C) Luohanzhe. IGS spacer was performed using a k-tuple of 8 with 100% identity.

To explore the nature of SR regions in the IGS in greater detail, we performed a bioinformatics analysis of the repeated DNA of IGS sequences using TRF and MDMP analysis. The TRF results showed a total of eight types of SRs in the IGS and different sizes in each SR (Fig 1; S4 and S5 Tables). Based on the sequence length, these SRs were classified into two categories: short SR and long SR. Five relatively short SRs (SR1, SR3, SR4, SR5, and SR6) were 24–32 bp in length, while the other three relatively long ones (SR2, SR7, and SR8) were 110–172 bp in length (S4 Table). Of these, the SR8 was the longest at 172 bp, whereas SR5 was the shortest with only 22 bp. Six SR blocks (SR1-SR6) occurred upstream of the TIS and two SR blocks (SR7 and SR8) were located downstream of the TIS (Fig 1). However, no SR element in *M*. *sinensis*, *Lycopersicon esculentum*, and *Zea mays* was present in the region downstream of the TIS, but the larger number of SRs was compensated upstream of the TIS [44, 49, 50]. At the intra- and the inter-species levels of the genus *Saccharum*, comparison results among the differently sized IGS variants revealed that the copy number of each types of SRs was similar, except for SR8, which contained a relatively higher copy number in *S*. *spontaneum* (Fig 1; S5 Table). These variants clearly occurred as a result of copy number variations in SR8. Among the three species, all SRs were highly conserved, and the length and sequence identity differed slightly among different SRs. Four SRs, including SR1 and SR2 at the beginning of the IGS and SR7 and SR8 at the end of the IGS, shared high similarity of at least 92.21%. With the exception of SR6, which shared comparatively low sequence similarity of 75%, the other three SRs (SR3, SR4, and SR5) had a mean similarity value of 88.71% (S4 Table). SR5 had the lowest GC content of 47.6%, but the other seven SRs exhibited high GC content at more than 60%, with SR7 having a GC content of 75% (S4 Table). Interestingly, SR7 and SR8 shared a partial overlapping sequence motif (Fig 1). A MDMP analysis was applied by self-comparison of the IGS sequences using LBDot. Blue dots represented the homolog regions of the IGS sequences both in the direct orientation, and red dots compared the IGS sequences in the direct orientation with in the reverse orientation. That is, blue dots represented the direct repeats, and red dots were representative for the inverted repeats. The distribution pattern of the direct and inverted repeats was shown by the concentrated distribution areas in the IGS sequences. The more the distribution density was, the more the copy number of the direct and inverted repeats was. Self-comparison analysis of three IGSs using MDMP further revealed that each spacer was composed of two intensively repeated regions, as shown in the square boxes in Fig 3. A significant number of direct repeats were concentrated in both regions, and the inverted repeats were scattered without a distinct distribution pattern in the IGS sequence. It is noteworthy that in the light of the distribution pattern, Luohanzhe and 51NG3 shared a similar distribution pattern of the direct and inverted repeats, whereas Yunnan82-114 was different. In addition, we found the presence of several base substitutions and partially deleted copies of each SR both at the individual level (data not shown) and at the inter-species level (S1 File).

### Phylogenetic relationship of the three original species based on analysis of IGS sequence

**Fig 4:**
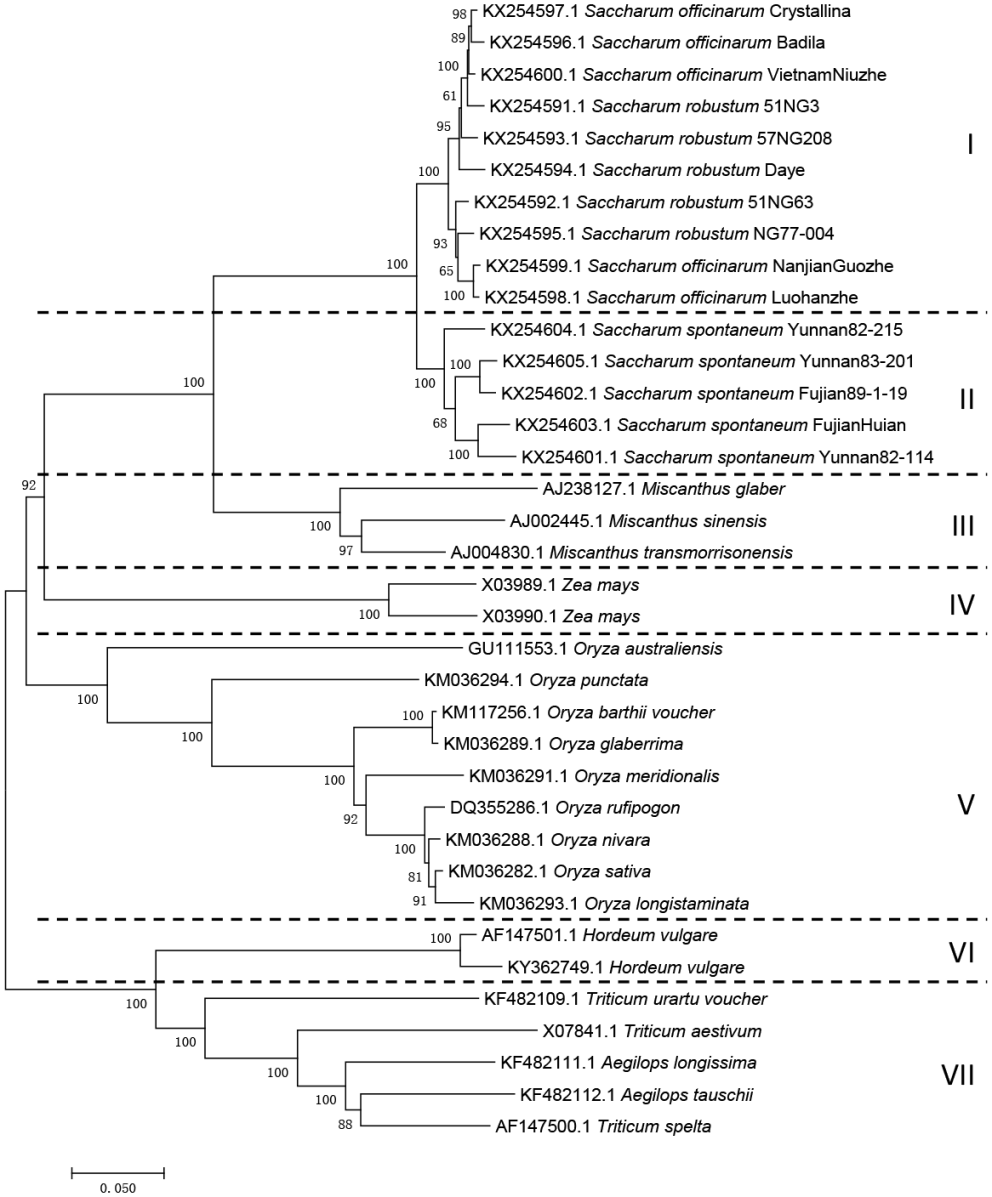
Phylogenetic relationships among 15 accessions of the genus *Saccharum*. Bootstrap values from 1000 tests are indicated at the nodes. The tree is drawn to scale, with branch lengths in the same units as those of the evolutionary distances used to infer the phylogenetic tree. The ML and NJ tree of the same data is topologically identical.

The phylogenetic trees were constructed using ML and NJ based on the IGS sequences. The cluster analysis revealed that the NJ tree was similar to the ML tree with seven major clusters for the three original species in the genus *Saccharum* and other species from the family *Poaceae*, including *Miscanthus*, *Zea*, *Oryza*, *Hordeum*, *Aegilops*, and *Triticum* (Fig 4). In the genus *Saccharum*, the five *S*. *spontaneum* fell into a single cluster (clade II), and all of them shared the same array of the SR8 element in terms of number and position. However, ten accessions of *S*. *robustum* and *S*. *officinarum* formed two discrete groups (clade I), and all of them were completely intermingled without any hint of distinct lineage between *S*. *robustum* and *S*. *officinarum*. This was simiar to the species from the genus *Triticum* and *Aegilops* (clade VII).

### Physical mapping of IGS in *S*. *spontaneum*, *S*. *robustum*, and *S*. *officinarum*

**Fig 5:**
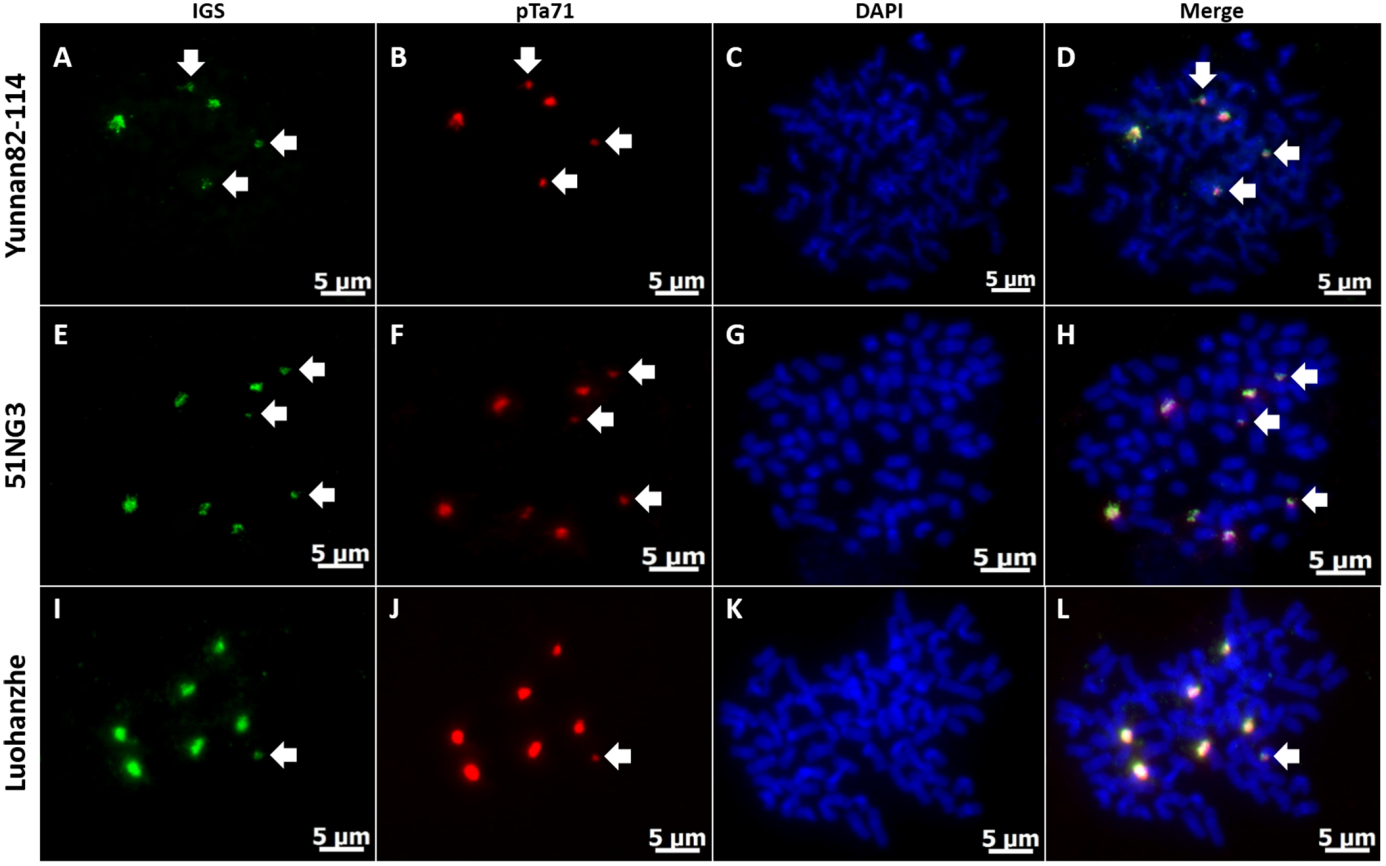
Physical mapping of IGS and pTa71 probe in Yunnan82-114 (*S*. *spontaneum*), 51NG3 (*S*. *robustum*) and Luohanzhe (*S*. *officinarum*). FISH with IGS (red- B, F, J), and wheat rDNA pTa71 probe (green- A, E, I) in meristematic root tip metaphase chromosomes of Yunnan82-114 (A-D), 51NG3 (E-H), and Luohanzhe (I-L). DNA is counterstained with DAPI (blue—C, G, K). The fourth column shows the merged images of both signals of IGS and wheat rDNA pTa71 probe and DAPI-stained chromosomes (D, H, L). Arrows indicate minor loci NORs (A, B, D; E, F, H; I, J, L). Scale bars = 5 μm.

To determine the chromosomal location between 45S rDNA and IGS within the three original species, including *S*. *spontaneum*, *S*. *robustum*, and *S*. *officinarum*, we selected one genotype in each original species as representatives, including Yunnan82-114 (*S*. *spontaneum*), 51NG3 (*S*. *robustum*), and Luohanzhe (*S*. *officinarum*). FISH analysis was applied simultaneously using the pTa71 probe for the 45S rDNA locus and IGS probe for the IGSs from each species on the metaphase chromosome. As expected, the IGSs from each species co-localized with the 45S rDNA, and the number of IGS signals was in accordance with the number of the 45S rDNA signals in each species. However, the number of loci detected varied, as follows: five loci in Yunnan82-114, seven loci in Luohanzhe, and eight loci in 51NG3 (Fig 5). In addition, the location of both 45S rDNA and the IGS in *S*. *spontaneum* can differ from that in *S*. *robustum* and *S*. *officinarum*. In agreemeent with the report of D'Hont et al. [6], the interstitial signal was constantly observed on the 45S rDNA-bearing chromosome in *S*. *spontaneum*, whereas the terminal signal was in *S*. *robustum* and *S*. *officinarum* (Fig 5). Remarkably, some minor loci NORs were found among Luohanzhe, 51NG3, and Yunnan82-114, but the number of minor loci NORs varied slightly. Of these, 51NG3 and Yunnan82-114 bore three minor loci NORs, while Luohanzhe carried only one minor locus NOR.

## Discussion

### Length heterogeneity at the individual, intraspecies, and interspecies levels of the genus *Saccharum*

In this study, we observed variable length of the IGS sequence at the individual, intraspecies, and interspecies levels of the genus *Saccharum*, including *S*. *spontaneum*, *S*. *robustum*, and *S*. *officinarum*. The IGS is a rapidly evolving region of the 45S rDNA with several internal SRs. The length of polymorphisms has been observed among populations, species, and even in individuals in many plant species [24]. For instance, Jo et al. found that the length of the IGS sequence differed greatly at the inter-species and inter-generic level of the family *Solanaceae* [51]. In contrast, we detected a narrow range of length variability at the individual, intraspecies, and interspecies levels of the genus *Saccharum*. Notably, our sequence analysis showed that the IGS variants occur mainly due to duplications or deletions and base substitutions at these three levels. Except for the duplications or deletions, the degree of sequence similarity was considerably high. The common characteristic within the rDNA IGS is the presence of SR elements for both low and high eukaryotes. In fact, heterogeneity of IGS length is attributed to duplications or deletions of the SR region that can be present in different copy numbers in almost all species [51–55]. Similarly, in this study, the variation in IGS length was due to variation in the copy number of one or several SR motifs generated by duplications or deletions. In other words, indels were restricted to SR-rich regions, suggesting that these elements are involved in the differences in length observed. The SR regions may be the hot-spot regions of the IGS during their evolutionary history. The polymorphism of IGS length is also believed to be the result of concerted evolution [15]. In plants, heterogeneity in the length of the IGS has been thought to be due to variations in the SR elements generated by unequal crossing over [19]. In this study, from the analysis between the different IGS variants in the intraindividual level, we found evidence that duplication and deletion events in the IGS region within the genus *Saccharum* gave rise to the differences in length as well as different copy numbers of SRs. The polymorphism of the IGS sequence may have arisen following seven indel events, except for the SR7 with the same copy number (3.7). As one of the major driving forces in the evolution of the rDNA units, this variability can be attributed to unequal crossing over between SR elements.

### Structure of promoter and terminator sequences and ETS region

Based on sequence analyses, we found several functional elements within the IGS sequences. The functional elements with high nucleotide conservation are hypothesized to be associated with the crucial process of initiating and terminating rRNA transcription by RNA polymerase I, such as a promoter, enhancer elements, and the TATA box. Based on comparisons with the promoter regions from other species, we only detected a unique putative promoter in the IGS variants of the genus *Saccharum*, although several animal and plant species have more than one promoter and the functional significance has been related to their transcriptional activity. Similar to the IGS characterized for most vascular plant species, the promoter region appears to be located between the repetitive areas (between SR6 and SR7) in the genus *Saccharum*. The putative TIS sequence (TATATT**A**GGGG) in the genus *Saccharum* contained the core sequence around the highly conserved A residue. The putative TIS sequence showed resemblance to the hypothesized consensus promoter sequence reported for the plant promoters. In most families of *Angiosperms*, the TATA and GGGG boxes in the core promoter seem to be a pervasive feature of rRNA transcription [24]. This also suggests that, rather than the sequence itself, a structural code is the determinant for its function, probably by directing specific DNA-protein interactions involved in transcriptional control. Similar to the GGGG boxes in most families of *Angiosperms*, the hexanucleotide sequence GGCGGG was previously found at similar regions in several eukaryotic promoters and was shown to be crucial for promoter activity [56]. In *P*. *granatum*, *M*. *sinensis*, *F*. *sylvatica*, *Q*. *suber*, *Q*. *petraea*, and *Q*. *robur*, the TATA is missing in the upstream of the conserved A residue of the putative TIS sequence [23, 43, 44]. Precise transcription factor interactions, dictated by the correct spacing of promoter elements, are key to promoter recognition, while TATA sequence may not be a direct binding site for transcription factors, but is conserved probably only for DNA to be easily melted [57]. In addition, it has been noted that in several plant species, the promoter sequences in IGS regions are usually duplicated or contain multiple repeats that enhance the polymerase activity via an increase in transcription initiation.

A unique AT-rich region upstream of the TIS in the genus *Saccharum* may represent potential binding protein regions involved in the TIS. The pyrimidine-rich motif sequence (CCCTCCCCCCCTCC) located just after the 3′ terminus of the 25S rRNA gene likely acts as a proximal terminator. The putative terminator was highly similar to a portion of the conserved motif present at the beginning of the IGS of some plant species. In general, the AT-rich sequences in several plants are preceded by a region composed of the putative TIS. This region could form the domain of the promoter sequence known as the upstream binding element, which confers species specificity to polymerase I [24, 43]. The presence of a short AT-rich region in the genus *Saccharum* has been shown to be associated with enhanced transcription.

In this study, the sequences of the NTS showed high divergence among *S*. *spontaneum*, *S*. *robustum*, and *S*. *officinarum*. In contrast to the lower level of similarity of the NTS region, the ETS region has better-conserved nucleotide sequences than the NTS region. Similar results were also observed in other grasses, such as *M*. *sinensis*, *Oryza sativa*, *Zea mays*, and *Triticum aestivum* [44, 49, 58, 59]. In addition, sequence alignment showed that the difference in ETS length among *S*. *spontaneum*, *S*. *robustum*, and *S*. *officinarum* was due to insertions of SR8, suggesting that SR8 might be an evolutionary hot-spot region in the IGS sequence. The result seemed to show that the length of IGS tended to reduce gradually from the most primitive species *S*. *spontaneum* to the domesticated species *S*. *officinarum* with increasing evolutionary relationship distance.

### CpG islands and methylation sites

The average GC content of the entire IGS region was over 60% in the genus *Saccharum*. The regions with the greatest GC content shared the SR and ETS regions in *Poaceae*. Indeed, there were two CpG islands in the entire IGS. A relatively small CpG island was located between the TIS and 3′ end of the 18S rRNA gene. As a rule, CpG islands have a GC content that is significantly higher than that of the genome average and are nonmethylated [60]. Cytosine is a DNA base that can be chemically modified, and cytosine methylation of CpG base pairs in plants is associated with the inhibition of rRNA gene transcription initiation [61]. Similar to *Avena*, *Olea*, and *Triticum* [46–48], the IGS of the genus *Saccharum* has a sufficient number of methylation sites that are irregularly distributed along the sequence. Indeed, methylation levels have been found to be associated with the transcription of rRNA genes [22, 62].

### Phylogenetic relationship of the three original species within the genus *Saccharum*

The spacers (45S rDNA ITS, IGS, and 5S rDNA NTS) with the higher variation in length and sequence allow for us to resolve the phylogenetic relationships of species. Phylogenetic relationships of inter-species in the genus *Saccharum* have been previously investigated with regard to sequence spacing. Liu et al. reported that *S*. *spontaneum*, *S*. *robustum*, and *S*. *officinarum* were clustered together based on 45S rDNA-ITS haplotype data in *S*. *spontaneum* [3]. Pan et al. found that the two *S*. *officinarum* accessions did not form distinct clusters, but rather were interrelated within the *S*. *spontaneum* cluster based on the 5S rDNA NTS sequences [4].

Phylogeny based on 45S rDNA ITS and 5S rDNA NTS sequences is clearly not enough to reflect the relationship of species within the genus *Saccharum*. The most plausible explanation is the lack of sufficient abundant variances in the highly conserved length of 45S rDNA ITS and 5S rDNA NTS sequences in the species of the genus *Saccharum*. The IGS region is evolving rapidly compared to the 45S rDNA ITS and 5S rDNA NTS sequences. In most plants, due to the differences in the SR sequences (including copy number, sequence, and length) of the IGS region among different species, the IGS region may provide a more suitable marker for reconstructing phylogenetic relationships within both inter- and intra-species than the 45 rDNA ITS and 5S rDNA NTS.

In this study, *S*. *officinarum* shared the same clade with *S*. *robustum* in the NJ tree, supporting the close relationship between *S*. *officinarum* and *S*. *robustum*. On the contrary, *S*. *spontaneum* formed a highly supported separate clade, indicating its phylogenetic isolation from *S*. *officinarum* and *S*. *robustum*. The phylogenetic relationship of the IGS sequences closely reflected the relationship of the species in the genus *Saccharum*. Many molecular marker studies have suggested that *S*. *robustum* is the progenitor of *S*. *officinarum*, while *S*. *spontaneum* is the most primitive species [6].

### FISH mapping IGS sequences are only located at the NOR

In all of the species of the genus *Saccharum* that were analyzed, the IGS always co-localized in the same loci with 45S rDNA, suggesting that IGS sequences were only located at the NOR. However, Jo et al. reported that in addition to the co-localization of 45S rDNA and IGS at the same loci, an independent IGS-derived satellite DNA was located outside the 45S rDNA loci in the *Capsicum* species [51]. Additionally, Chester et al. reported that the IGS sequences containing a truncated retrotransposon sequence similar to members of the Copia superfamily was dispersed along both pairs of 45S rDNA loci [63]. Theoretically, the number of rDNA loci should be equal to the basic chromosome numbers in autopolyploid of the family *Poaceae*. For instance, the diploid species, such as *Zea mays* and *Sorghum bicolor*, have two 5S rDNA loci as well as two 45S rDNA loci [64, 65]. In this study, the decaploid species Yunnan82-114 (*S*. *spontaneum*) contained five loci, whereas the two octaploid species Luohanzhe (*S*. *officinarum*) and 51NG3 (*S*. *robustum*) bore seven and eight loci, respectively. Except for 51NG3 (*S*. *robustum*), the difference between the expected and observed number of 45S rDNA loci was present in Yunnan82-114 (*S*. *spontaneum*) and Luohanzhe (*S*. *officinarum*). This result suggests that the 45S rDNA loci in the genus *Saccharum* seems to experience a dynamic evolution and some 45S rDNA loci appear to have been eliminated. Indeed, chromosomal polymorphisms in the number and chromosomal position of the 45S rDNA loci were also found in other grasses, such as *Oryza* and *Paspalum* [66, 67]. In contrast to the 45S rDNA loci, the 5S rDNA loci are usually not syntenic to the 45S rDNA and are generally distributed in an interstitial position in the chromosomes among most species, suggesting that the location of the 5S rDNA sequence appears to be conserved. Such a conserved interstitial position within the 5S rDNA loci does not appear to result in the elimination of 5S rDNA. Thus, compared with 45S rDNA, 5S rDNA is more suitable for determining the basic chromosome numbers of species. For the three original species of the genus *Saccharum*, D'Hont et al. also provided evidence that the number of 5S rDNA loci corresponded well with the ploidy level, but the number of 45S rDNA sites was variable [6]. Notably, the distribution of co-localized loci was different among *S*. *spontaneum*, *S*. *robustum*, and *S*. *officinarum*. The co-localized loci were located in terminal regions of chromosomes in *S*. *officinarum* and *S*. *robustum*, whereas they were located more interstitially in *S*. *spontaneum*. A similar pattern was observed by D'Hont et al., who found that 45S rDNA sites were terminal for *S*. *officinarum* and *S*. *robustum* and interstitial for *S*. *spontaneum* [6]. Additionally, a BAC of 45S rDNA units (SCHRBa 039 D18) was obtained from the sugarcane hybrid R570, an amphidiploid derived from cross between *S*. *officinarum* and *S*. *spontaneum* [68]. Analyses of the sequence from the BAC of 45S rDNA units showed that there were 14 45S rDNA units with a portion of one unit in the reverse orientation to the other 13 and the sequence length of IGS is 3011 bp. The result of sequence alignment with our IGS sequences from the three species revealed that the IGS sequences from the BAC of 45S rDNA units are high simiar to the IGS sequences from our genotypes of *S*. *spontaneum*. Thus, the BAC of 45S rDNA units was derived from *S*. *spontaneum*. This BAC was located to metaphases from root tips of the sugarcane cultivar R570. FISH result revealed that there were the less intense interstitial rDNA signals and the more intense terminal signals, implying 45S rDNA-bearing chromosomes are derived from *S*. *officinarum* and *S*. *spontaneum* chromosomes in this sugarcane modern cultivar R570.

## Conclusion

In the present study, we performed for the first time a comparative analysis of the sequence organization of the IGS, and the chromosome locations of the IGS within these three original species. We found length variability of the IGS region in the three original species and a similar overall organization in the IGS as follows: (1) the presence of eight SR blocks (upstream and downstream from TIS); (2) a unique AT-rich region upstream of the TIS; (3) two CpG islands in NTS and ETS; (4) a highly homologous terminator and promoter regions with the same TTS and TIS sequences and other regulatory elements; and (5) an ETS with high similarity among species and an NTS with relatively higher variability. Moreover, the location of the IGS regions exclusively at the NORs was determined for different original species of the genus *Saccharum*. The results of this study provide important insights into the structure and organization of the IGS in the genus *Saccharum*.

## Supporting information

S1 File Sequence alignment of the sub-repeats of 15 genotypes at the inter-species level of the genus *Saccharum*. (PDF)

S1 Table The internal primer used for sequencing of IGS sequences. (DOCX)

S2 Table IGS characterization in 15 genotypes of *the genus Saccharum*. (DOCX)

S3 Table The CpG islands location in the IGS region. (DOCX)

S4 Table Sub-repeat in the genus *Saccharum*. (DOCX)

S5 Table The copy number of SR in the genus *Saccharum*. (DOCX)
