# Supplementary material for: Comparative genetic analysis of the 45S rDNA intergenic spacers from three Saccharum species
Source: PLoS One. 2017 Aug 17;12(8):e0183447. doi: 10.1371/journal.pone.0183447 (PMC5560572; doi:10.1371/journal.pone.0183447)
Supplement: S1 Table — (DOCX) [file pone.0183447.s002.docx]

| **No.** | **Accession** | **Species** | | **Primer** | | **Sequence (5'→3')** | |
| --- | --- | --- | --- | --- | --- | --- | --- |
| 1 | Yunnan82-215 | *S. spontaneum* | | D1114-112-71799J-R3 | | CACCTCGATGCGCGTATGAA | |
|  |  |  | | D1209-109-76058J-F2 | | GCCGGGAGTCGTTCCGACAA | |
|  |  |  | | D1202-161-76058J-F1 | | GCAACCGAGGGTCCACAGAG | |
| 2 | Yunna83-201 | *S. spontaneum* | | D1220-118-74772J-R1 | | AACCGAGGGTCCACAGAGCG | |
|  |  |  | | D1223-106-74772J-R2 | | CCGGCGCATCATCAAAACAG | |
|  |  |  | | D1225-118-74772J-R3 | | GTTTTCTGTATGGTTCCTTC | |
| 3 | Yunnan82-114 | *S. spontaneum* | | D1220-118-74772J-R1 | | AACCGAGGGTCCACAGAGCG | |
|  |  |  | | D1223-106-74772J-R2 | | CCGGCGCATCATCAAAACAG | |
|  |  |  | | D1225-118-74772J-R3 | | GTTTTCTGTATGGTTCCTTC | |
| 4 | Fujian Huian | *S. spontaneum* | | D1209-108-76053J-F2 | | CAACACGTTGGCCACCCCAG | |
|  |  |  | | D1202-161-76058J-F1 | | GCAACCGAGGGTCCACAGAG | |
|  |  |  | | D1209-112-76071J-R3 | | AGCCCCCCCAGGAGTTCTTG | |
| 5 | Fujian89-1-19 | *S. spontaneum* | | D1220-117-74772J-F1 | | GCAGTTCGGCAAGGCGGTCC | |
|  |  |  | | D1220-118-74772J-R1 | | AACCGAGGGTCCACAGAGCG | |
|  |  |  | | D1223-106-74772J-R2 | | CCGGCGCATCATCAAAACAG | |
| 6 | 51NG3 | *S. robustum* | | D1118-109-72567J-F2 | | AACCGAGGGTCCACAGAG | |
|  |  |  | | D1116-355-72567J-F1 | | TTCTCCCAGTGCGGTCACTTA | |
| 7 | 57NG208 | *S. robustum* | | D1112-215-71101J-R3 | | CCTTTCTTGGCTCGTT | |
|  |  |  | | D1110-52-71099J-R2 | | CAGCCACGAAGGTCAGG | |
|  |  |  | | D1108-52-71101J-R1 | | AACCGAGGGTCCACAGAG | |
| 8 | Daye | *S. robustum* | | D1220-118-74772J-R1 | | AACCGAGGGTCCACAGAGCG | |
|  |  |  | | D1225-119-74784J-R3 | | GACTTGGGCTGCTCCTTCTC | |
|  |  |  | | D1223-107-74784J-R2 | | CAGTGCGGTCACTTAGCATG | |
| 9 | 51NG63 | *S. robustum* | | E0407-357-79800J-F2 | | AAACTTCTCCCAGTGCGG | |
|  |  |  | | E0406-353-79800J-R1 | | GGTCCGTGGAAGACAGGC | |
|  |  |  | | E0402-353-79800J-F1 | | AACCGAGGGTCCACAGAG | |
| 10 | NG77-004 | *S. robustum* | | E0402-353-79800J-F1 | | AACCGAGGGTCCACAGAG | |
|  |  |  | | E0407-357-79800J-F2 | | AAACTTCTCCCAGTGCGG | |
|  |  |  | | E0406-353-79800J-R1 | | GGTCCGTGGAAGACAGGC | |
| 11 | Badila | | *S. officinarum* | | D1225-119-74784J-R3 | | GACTTGGGCTGCTCCTTCTC |
|  |  | |  | | D1220-118-74772J-R1 | | AACCGAGGGTCCACAGAGCG |
|  |  | |  | | D1223-107-74784J-R2 | | CAGTGCGGTCACTTAGCATG |
| 12 | Nanjian Guozhe | | *S. officinarum* | | D1208-113-CK-R2 | | CGGGGGATGCGTGACCGACTT |
|  |  | |  | | D1208-112-70713J-R1 | | CGTTCCAGCAACAAGATTCC |
| 13 | Crystallina | | *S. officinarum* | | D1208-113-CK-R2 | | CGGGGGATGCGTGACCGACTT |
|  |  | |  | | D1208-112-70713J-R1 | | CGTTCCAGCAACAAGATTCC |
| 14 | Luohanzhe | | *S. officinarum* | | D1208-112-70713J-R1 | | CGTTCCAGCAACAAGATTCC |
|  |  | |  | | D1208-113-CK-R2 | | CGGGGGATGCGTGACCGACTT |
| 15 | Vietnam Niuzhe | | *S. officinarum* | | D1208-113-CK-R2 | | CGGGGGATGCGTGACCGACTT |
|  |  | |  | | D1208-112-70713J-R1 | | CGTTCCAGCAACAAGATTCC |
