# Supplementary material for: Comparative genetic analysis of the 45S rDNA intergenic spacers from three Saccharum species
Source: PLoS One. 2017 Aug 17;12(8):e0183447. doi: 10.1371/journal.pone.0183447 (PMC5560572; doi:10.1371/journal.pone.0183447)
Supplement: S2 Table — (DOCX) [file pone.0183447.s003.docx]

| **No.** | **Accession** | **Species** | **IGS** | |
| --- | --- | --- | --- | --- |
|  |  |  | **Length (bp)** | **GC content (%)** |
| 1 | Yunnan82-215 | *S. spontaneum* | 2783 | 64.2 |
|  |  |  | 2788 | 65.9 |
|  |  |  | 2796 | 64.5 |
|  |  |  | 2805 | 65.1 |
|  |  |  | 2819 | 64.9 |
|  |  |  | 2833 | 65.0 |
|  |  |  | 2865 | 64.4 |
|  |  |  | 2898 | 64.7 |
| 2 | Yunnan83-201 | *S. spontaneum* | 2946 | 64.2 |
|  |  |  | 2950 | 63.6 |
|  |  |  | 3045 | 63.4 |
|  |  |  | 3078 | 64.5 |
|  |  |  | 3098 | 65.0 |
|  |  |  | 3101 | 65.1 |
|  |  |  | 3117 | 63.8 |
|  |  |  | 3185 | 64.2 |
| 3 | Yunnan82-114 | *S. spontaneum* | 3185 | 64.6 |
|  |  |  | 3213 | 64.5 |
|  |  |  | 3254 | 64.9 |
|  |  |  | 3297 | 65.0 |
|  |  |  | 3305 | 64.8 |
|  |  |  | 3321 | 63.9 |
|  |  |  | 3334 | 64.9 |
|  |  |  | 3340 | 63.3 |
| 4 | Fujian Huian | *S. spontaneum* | 2821 | 64.2 |
|  |  |  | 2829 | 64.0 |
|  |  |  | 2835 | 63.9 |
|  |  |  | 2866 | 64.2 |
|  |  |  | 2905 | 64.8 |
|  |  |  | 2978 | 64.2 |
|  |  |  | 3071 | 64.0 |
|  |  |  | 3108 | 63.8 |
| 5 | Fujian89-1-19 | *S. spontaneum* | 3109 | 63.8 |
|  |  |  | 3118 | 63.9 |
|  |  |  | 3158 | 64.3 |
|  |  |  | 3187 | 64.1 |
|  |  |  | 3213 | 64.2 |
|  |  |  | 3265 | 64.9 |
|  |  |  | 3278 | 63.8 |
|  |  |  | 3317 | 64.6 |

Continued from previous table

| **No.** | **Accession** | **Species** | **IGS** | |
| --- | --- | --- | --- | --- |
|  |  |  | **Length (bp)** | **GC content (%)** |
| 6 | Daye | *S. robustum* | 2728 | 65.7 |
|  |  |  | 2735 | 66.4 |
|  |  |  | 2739 | 66.4 |
|  |  |  | 2746 | 65.9 |
|  |  |  | 2750 | 66.1 |
|  |  |  | 2753 | 65.9 |
|  |  |  | 2758 | 65.4 |
|  |  |  | 2766 | 65.1 |
| 7 | 51NG63 | *S. robustum* | 2592 | 65.0 |
|  |  |  | 2697 | 66.0 |
|  |  |  | 2702 | 66.3 |
|  |  |  | 2709 | 65.9 |
|  |  |  | 2711 | 65.7 |
|  |  |  | 2720 | 66.1 |
|  |  |  | 2728 | 65.7 |
|  |  |  | 2734 | 66.0 |
| 8 | 51NG3 | *S. robustum* | 2718 | 65.9 |
|  |  |  | 2721 | 65.8 |
|  |  |  | 2727 | 65.8 |
|  |  |  | 2735 | 66.0 |
|  |  |  | 2778 | 66.2 |
|  |  |  | 2820 | 66.0 |
|  |  |  | 2842 | 66.2 |
|  |  |  | 2862 | 66.1 |
| 9 | 57NG208 | *S. robustum* | 2631 | 64.7 |
|  |  |  | 2635 | 63.8 |
|  |  |  | 2659 | 66.1 |
|  |  |  | 2677 | 65.9 |
|  |  |  | 2716 | 66.0 |
|  |  |  | 2722 | 65.8 |
|  |  |  | 2728 | 66.1 |
|  |  |  | 2856 | 66.3 |
| 10 | NG77-004 | *S. robustum* | 2691 | 66.0 |
|  |  |  | 2699 | 66.2 |
|  |  |  | 2715 | 66.1 |
|  |  |  | 2721 | 66.1 |
|  |  |  | 2727 | 65.9 |
|  |  |  | 2739 | 66.0 |
|  |  |  | 2801 | 66.4 |
|  |  |  | 2845 | 66.6 |

Continued from previous table

| **No.** | **Accession** | **Species** | **IGS** | |
| --- | --- | --- | --- | --- |
|  |  |  | **Length (bp)** | **GC content (%)** |
| 11 | Badila | *S. officinarum* | 2593 | 65.4 |
|  |  |  | 2604 | 65.1 |
|  |  |  | 2611 | 65.0 |
|  |  |  | 2623 | 66.1 |
|  |  |  | 2649 | 65.3 |
|  |  |  | 2671 | 66.0 |
|  |  |  | 2698 | 66.2 |
|  |  |  | 2730 | 65.4 |
| 12 | Nanjian Guozhe | *S. officinarum* | 2592 | 65.5 |
|  |  |  | 2599 | 65.9 |
|  |  |  | 2610 | 65.8 |
|  |  |  | 2634 | 66.0 |
|  |  |  | 2679 | 65.7 |
|  |  |  | 2725 | 65.7 |
|  |  |  | 2731 | 65.4 |
|  |  |  | 2736 | 65.9 |
| 13 | Vietnam Niuzhe | *S. officinarum* | 2681 | 66.1 |
|  |  |  | 2699 | 65.9 |
|  |  |  | 2719 | 65.9 |
|  |  |  | 2723 | 65.4 |
|  |  |  | 2725 | 65.8 |
|  |  |  | 2728 | 65.9 |
|  |  |  | 2731 | 65.9 |
|  |  |  | 2736 | 65.8 |
| 14 | Crystallina | *S. officinarum* | 2651 | 65.6 |
|  |  |  | 2664 | 65.1 |
|  |  |  | 2677 | 66.0 |
|  |  |  | 2679 | 65.9 |
|  |  |  | 2683 | 66.2 |
|  |  |  | 2698 | 65.8 |
|  |  |  | 2710 | 66.2 |
|  |  |  | 2725 | 65.7 |
| 15 | Luohanzhe | *S. officinarum* | 2590 | 65.2 |
|  |  |  | 2623 | 65.6 |
|  |  |  | 2632 | 65.2 |
|  |  |  | 2639 | 65.1 |
|  |  |  | 2651 | 65.9 |
|  |  |  | 2694 | 65.9 |
|  |  |  | 2725 | 65.8 |
|  |  |  | 2737 | 66.1 |
