# Supplementary material for: Comparative genetic analysis of the 45S rDNA intergenic spacers from three Saccharum species
Source: PLoS One. 2017 Aug 17;12(8):e0183447. doi: 10.1371/journal.pone.0183447 (PMC5560572; doi:10.1371/journal.pone.0183447)
Supplement: S3 Table — (DOCX) [file pone.0183447.s004.docx]

| **Accession** | **Species** | **CpG island** | | | | | | |
| --- | --- | --- | --- | --- | --- | --- | --- | --- |
|  |  | **NTS** | | |  | **ETS** | | |
|  |  | **Length (bp)** | **Position** | **GC (%)** |  | **Length (bp)** | **Position** | **GC(%)** |
| Yunnan82-215 | *S. spontaneum* | 1003 | 129 ~1131 | 67.6 |  | 534 | 933 ~ 1466 | 60.8 |
| Yunnan83-201 | *S. spontaneum* | 1029 | 129 ~ 1157 | 67.6 |  | 560 | 1202 ~ 1761 | 59.8 |
| Yunnan82-114 | *S. spontaneum* | 670 | 136 ~ 805 | 61.9 |  | 548 | 1379~ 1926 | 59.5 |
| Fujian Huian | *S. spontaneum* | 921 | 49~ 1071 | 66.9 |  | 569 | 757 ~ 1302 | 60.7 |
| Fujian89-1-19 | *S. spontaneum* | 1135 | 128 ~ 1262 | 67.9 |  | 577 | 1178~ 1744 | 60.8 |
| 51NG3 | *S. robustum* | 1248 | 48 ~ 1295 | 68.7 |  | 544 | 750 ~ 1294 | 61.8 |
| 57NG208 | *S. robustum* | 1241 | 48 ~ 1288 | 69.5 |  | 545 | 750 ~ 1294 | 61.1 |
| Daye | *S. robustum* | 1115 | 48 ~ 1162 | 67.1 |  | 546 | 751 ~ 1296 | 61.6 |
| 51NG63 | *S. robustum* | 1120 | 47~1166 | 69.7 |  | 628 | 667 ~ 1294 | 61.5 |
| NG77-004 | *S. robustum* | 1230 | 48~1277 | 70.4 |  | 735 | 744 ~ 1295 | 62.5 |
| Badila | *S. officinarum* | 1086 | 48 ~ 1133 | 68.1 |  | 545 | 750 ~ 1294 | 60.8 |
| Nanjian Guozhe | *S. officinarum* | 1121 | 48 ~ 1168 | 69.9 |  | 628 | 667~ 1294 | 61.2 |
| Luohanzhe | *S. officinarum* | 1122 | 48 ~ 1169 | 69.1 |  | 579 | 718~ 1296 | 61.8 |
| Crystallina | *S. officinarum* | 1110 | 48 ~ 1157 | 68.4 |  | 549 | 747 ~ 1295 | 61.2 |
| Vietnam Niuzhe | *S. officinarum* | 1123 | 48 ~ 1170 | 68.4 |  | 546 | 750 ~ 1295 | 61.6 |
