# Supplementary material for: Comparative genetic analysis of the 45S rDNA intergenic spacers from three Saccharum species
Source: PLoS One. 2017 Aug 17;12(8):e0183447. doi: 10.1371/journal.pone.0183447 (PMC5560572; doi:10.1371/journal.pone.0183447)
Supplement: S4 Table — (DOCX) [file pone.0183447.s005.docx]

| **Sub-repeat** | **Identity (%)** | **Length (bp)** | **GC content (%)** |
| --- | --- | --- | --- |
| SR1 | 97.77 | 32 | 65.6 |
| SR2 | 95.77 | 135 | 74.8 |
| SR3 | 88.71 | 31 | 63.3 |
| SR4 | 88.14 | 24 | 66.6 |
| SR5 | 89.29 | 22 | 47.6 |
| SR6 | 75.00 | 32 | 75.0 |
| SR7 | 92.21 | 110 | 69.1 |
| SR8 | 94.68 | 172 | 61.6 |
