# Supplementary material for: Comparative genetic analysis of the 45S rDNA intergenic spacers from three Saccharum species
Source: PLoS One. 2017 Aug 17;12(8):e0183447. doi: 10.1371/journal.pone.0183447 (PMC5560572; doi:10.1371/journal.pone.0183447)
Supplement: S5 Table — (DOCX) [file pone.0183447.s006.docx]

| **Accession** | **Species** | **SR1** | **SR2** | **SR3** | **SR4** | **SR5** | **SR6** | **SR7** | **SR8** |
| --- | --- | --- | --- | --- | --- | --- | --- | --- | --- |
| Yunnan82-215 | *S. spontaneum* | 1.4 | 3.0 | 1.5 | 2.0 | 2.0 | 1.5 | 3.7 | 1.9 |
| Yunnan83-201 | *S. spontaneum* | 1.4 | 3.0 | 1.5 | 2.0 | 2.7 | 2.0 | 3.7 | 3.7 |
| Yunnan82-114 | *S. spontaneum* | 1.4 | 3.0 | 1.0 | 2.0 | 2.7 | 2.0 | 3.7 | 4.6 |
| Fujian Huian | *S. spontaneum* | 1.4 | 2.7 | 1.0 | 2.0 | 1.8 | 1.0 | 3.7 | 3.7 |
| Fujian89-1-19 | *S. spontaneum* | 1.4 | 4.0 | 1.8 | 2.0 | 2.7 | 2.0 | 3.7 | 3.7 |
| 51NG3 | *S. robustum* | 1.4 | 3.7 | 1.0 | 2.0 | 2.7 | 2.0 | 3.7 | 0.9 |
| 57NG208 | *S. robustum* | 2.0 | 3.7 | 1.0 | 2.0 | 2.4 | 2.0 | 3.7 | 0.9 |
| Daye | *S. robustum* | 2.0 | 2.7 | 1.0 | 2.0 | 2.7 | 1.0 | 3.7 | 0.9 |
| 51NG63 | *S. robustum* | 1.4 | 3.7 | 1.0 | 2.0 | 2.7 | 2.0 | 3.7 | 0.9 |
| NG77-004 | *S. robustum* | 1.4 | 4.7 | 1.0 | 1.0 | 2.7 | 2.0 | 3.7 | 0.9 |
| Badila | *S. officinarum* | 1.4 | 2.7 | 1.0 | 2.0 | 2.7 | 2.0 | 3.7 | 0.9 |
| Nanjian Guozhe | *S. officinarum* | 1.4 | 3.6 | 1.0 | 2.0 | 2.7 | 2.0 | 3.7 | 0.9 |
| Luohanzhe | *S. officinarum* | 1.4 | 3.7 | 1.0 | 3.0 | 2.7 | 2.0 | 3.7 | 0.9 |
| Crystallina | *S. officinarum* | 1.4 | 2.7 | 1.0 | 2.0 | 2.7 | 2.0 | 3.7 | 0.9 |
| Vietnam Niuzhe | *S. officinarum* | 1.4 | 2.7 | 1.0 | 2.0 | 2.7 | 2.0 | 3.7 | 0.9 |
