# Supplementary material for: Comparative genetic analysis of the 45S rDNA intergenic spacers from three Saccharum species
Source: PLoS One. 2017 Aug 17;12(8):e0183447. doi: 10.1371/journal.pone.0183447 (PMC5560572; doi:10.1371/journal.pone.0183447)
Supplement: S1 File — (PDF) [file pone.0183447.s001.pdf]

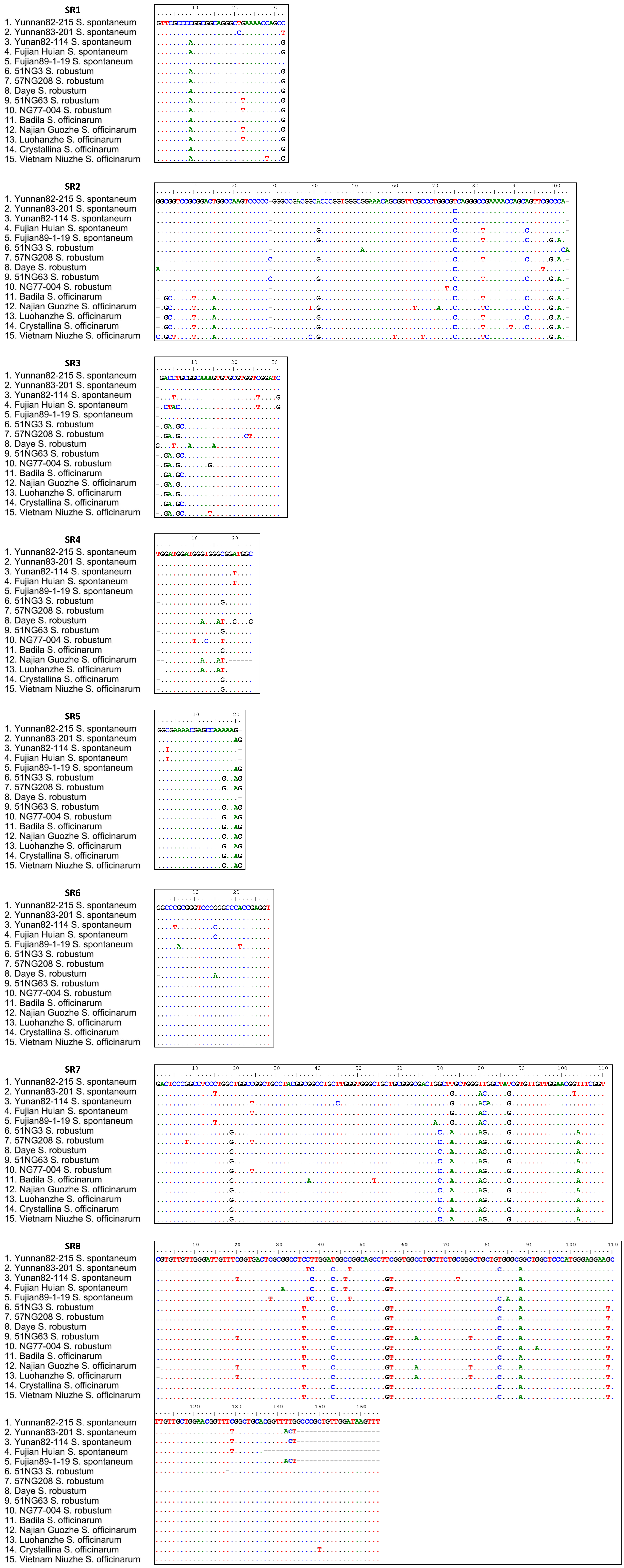
SR1
1. Yunnan82-215 S. spontaneum
2. Yunnan83-201 S. spontaneum
3. Yunan82-114 S. spontaneum
4. Fujian Huian S. spontaneum
5. Fujian89-1-19 S. spontaneum
6. 51NG3 S. robustum
7. 57NG208 S. robustum
8. Daye S. robustum
9. 51NG63 S. robustum
10. NG77-004 S. robustum
11. Badila S. officinarum
12. Najian Guozhe S. officinarum
13. Luohanzhe S. officinarum
14. Crystallina S. officinarum
15. Vietnam Niuzhe S. officinarum
SR2
1. Yunnan82-215 S. spontaneum
2. Yunnan83-201 S. spontaneum
3. Yunan82-114 S. spontaneum
4. Fujian Huian S. spontaneum
5. Fujian89-1-19 S. spontaneum
6. 51NG3 S. robustum
7. 57NG208 S. robustum
8. Daye S. robustum
9. 51NG63 S. robustum
10. NG77-004 S. robustum
11. Badila S. officinarum
12. Najian Guozhe S. officinarum
13. Luohanzhe S. officinarum
14. Crystallina S. officinarum
15. Vietnam Niuzhe S. officinarum
SR3
1. Yunnan82-215 S. spontaneum
2. Yunnan83-201 S. spontaneum
3. Yunan82-114 S. spontaneum
4. Fujian Huian S. spontaneum
5. Fujian89-1-19 S. spontaneum
6. 51NG3 S. robustum
7. 57NG208 S. robustum
8. Daye S. robustum
9. 51NG63 S. robustum
10. NG77-004 S. robustum
11. Badila S. officinarum
12. Najian Guozhe S. officinarum
13. Luohanzhe S. officinarum
14. Crystallina S. officinarum
15. Vietnam Niuzhe S. officinarum
SR4
1. Yunnan82-215 S. spontaneum
2. Yunnan83-201 S. spontaneum
3. Yunan82-114 S. spontaneum
4. Fujian Huian S. spontaneum
5. Fujian89-1-19 S. spontaneum
6. 51NG3 S. robustum
7. 57NG208 S. robustum
8. Daye S. robustum
9. 51NG63 S. robustum
10. NG77-004 S. robustum
11. Badila S. officinarum
12. Najian Guozhe S. officinarum
13. Luohanzhe S. officinarum
14. Crystallina S. officinarum
15. Vietnam Niuzhe S. officinarum
SR5
1. Yunnan82-215 S. spontaneum
2. Yunnan83-201 S. spontaneum
3. Yunan82-114 S. spontaneum
4. Fujian Huian S. spontaneum
5. Fujian89-1-19 S. spontaneum
6. 51NG3 S. robustum
7. 57NG208 S. robustum
8. Daye S. robustum
9. 51NG63 S. robustum
10. NG77-004 S. robustum
11. Badila S. officinarum
12. Najian Guozhe S. officinarum
13. Luohanzhe S. officinarum
14. Crystallina S. officinarum
15. Vietnam Niuzhe S. officinarum
SR6
1. Yunnan82-215 S. spontaneum
2. Yunnan83-201 S. spontaneum
3. Yunan82-114 S. spontaneum
4. Fujian Huian S. spontaneum
5. Fujian89-1-19 S. spontaneum
6. 51NG3 S. robustum
7. 57NG208 S. robustum
8. Daye S. robustum
9. 51NG63 S. robustum
10. NG77-004 S. robustum
11. Badila S. officinarum
12. Najian Guozhe S. officinarum
13. Luohanzhe S. officinarum
14. Crystallina S. officinarum
15. Vietnam Niuzhe S. officinarum
SR7
1. Yunnan82-215 S. spontaneum
2. Yunnan83-201 S. spontaneum
3. Yunan82-114 S. spontaneum
4. Fujian Huian S. spontaneum
5. Fujian89-1-19 S. spontaneum
6. 51NG3 S. robustum
7. 57NG208 S. robustum
8. Daye S. robustum
9. 51NG63 S. robustum
10. NG77-004 S. robustum
11. Badila S. officinarum
12. Najian Guozhe S. officinarum
13. Luohanzhe S. officinarum
14. Crystallina S. officinarum
15. Vietnam Niuzhe S. officinarum
SR8
1. Yunnan82-215 S. spontaneum
2. Yunnan83-201 S. spontaneum
3. Yunan82-114 S. spontaneum
4. Fujian Huian S. spontaneum
5. Fujian89-1-19 S. spontaneum
6. 51NG3 S. robustum
7. 57NG208 S. robustum
8. Daye S. robustum
9. 51NG63 S. robustum
10. NG77-004 S. robustum
11. Badila S. officinarum
12. Najian Guozhe S. officinarum
13. Luohanzhe S. officinarum
14. Crystallina S. officinarum
15. Vietnam Niuzhe S. officinarum
1. Yunnan82-215 S. spontaneum
2. Yunnan83-201 S. spontaneum
3. Yunan82-114 S. spontaneum
4. Fujian Huian S. spontaneum
5. Fujian89-1-19 S. spontaneum
6. 51NG3 S. robustum
7. 57NG208 S. robustum
8. Daye S. robustum
9. 51NG63 S. robustum
10. NG77-004 S. robustum
11. Badila S. officinarum
12. Najian Guozhe S. officinarum
13. Luohanzhe S. officinarum
14. Crystallina S. officinarum
15. Vietnam Niuzhe S. officinarum
